# Psychiatry Pitstop: Enhancing Communication Skills of Medical Students in Mental Health Settings

**DOI:** 10.1192/j.eurpsy.2024.388

**Published:** 2024-08-27

**Authors:** D. Magalhaes, F. Martinho, F. Viegas, M. Cativo, V. Ferreira, C. Manuel, S. Martins, J. Bastos, V. Barata, A. Pimentel, S. Carvalho, M. Santos, D. Almeida, L. Fernandes

**Affiliations:** ^1^Mental Health Department, Hospital Prof. Doutor Fernando Fonseca; ^2^Hospital Beatriz Ângelo, Lisbon, Portugal

## Abstract

**Introduction:**

*Psychiatry Pitstop* is a role-play-based program for medical students aimed to improve communication skills in the framework of mental health. The workshop involved amateur actors who simulated different clinical scenarios and psychiatry residents, who facilitated the sessions and provided constructive feedback following the Pendleton method. *Psychiatry Pitstop* was originally developed in the United Kingdom and it was expanded to Lisbon, Portugal, in 2019. The authors adapted the course to the Portuguese context, adjusting the number of sessions and altering the scenarios to match common clinical situations faced by junior doctors in Portugal. By now, we conducted four courses.

**Objectives:**

Our study aims to describe the Portuguese adaptation of the program and to learn insights from the students feedback.

**Methods:**

The course was assessed using satisfaction questionnaires, completed by the students after each session. These included a Likert scale ranging from 1 to 5, with items pertaining to Future Importance, Overall Quality, Theoretical Quality, and Practical Quality. Quantitative data was analyzed using Excel and standard descriptive statistics to summarize the results. The open questions invited students to articulate the main positive aspects, suggestions for improvement and future topics. A Natural Language Processing (NLP) software was used to evaluate open-ended responses and extract the main concepts.

**Results:**

We obtained a total of 39 single-answers from 4 different courses. Evaluation results yielded a mean score of 4.7 for Future Importance, 4.9 for Overall Quality, 4.3 for Theoretical Teaching, and 4.9 for Practical Teaching. Notable positive aspects included students’ appreciation of the immersive interview environment, the dedication exhibited by actors and doctors, well-prepared case scenarios, and engaging interactions with participants. Suggestions for improvement encompass enhanced theoretical introductions, comprehensive topic coverage, universal participation in simulations, and expanded workshop days. Future prospects for the program include practicing interviews with other psychiatric diagnosis, addressing difficult patients, delivering bad news and covering topics related to sexuality, grief and moral dilemmas.

**Conclusions:**

Our study shows that *Psychiatry Pitstop* adaptation to the Portuguese context was successful. Overall, the feedback from medical students has been consistently positive. Subsequent editions will draw upon the findings of this study to enhance overall program quality.

**Disclosure of Interest:**

None Declared

